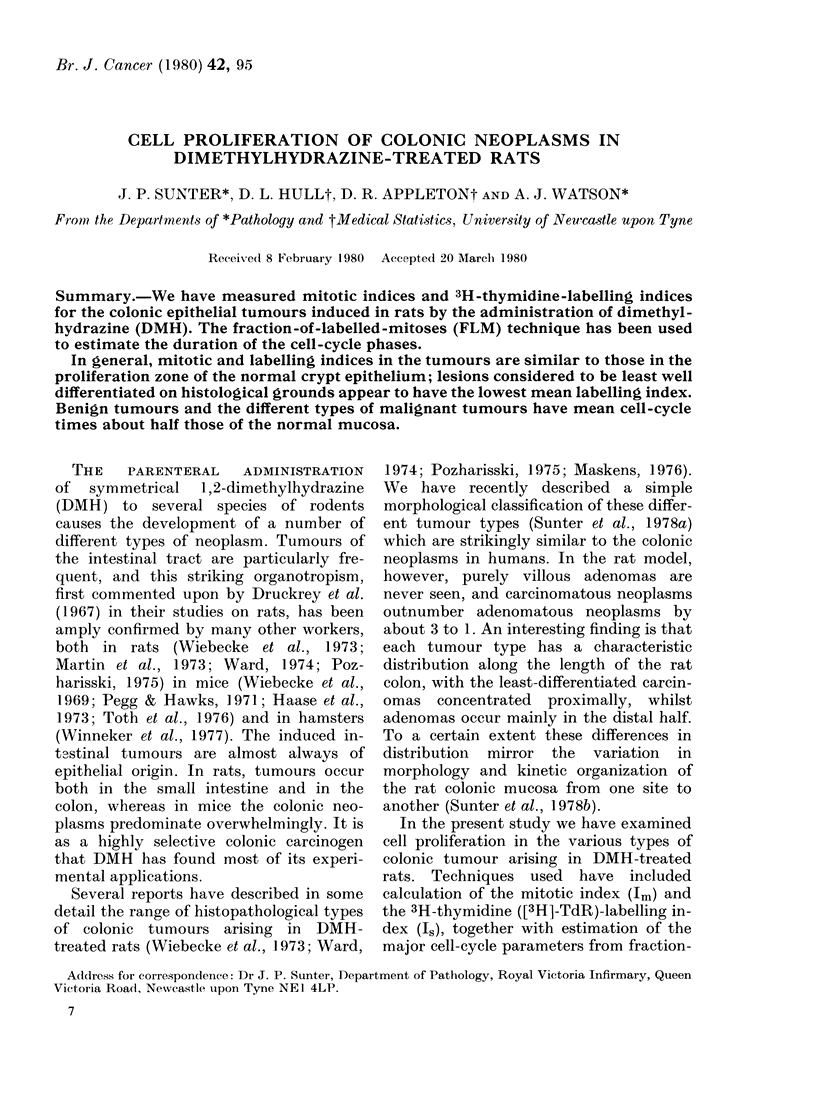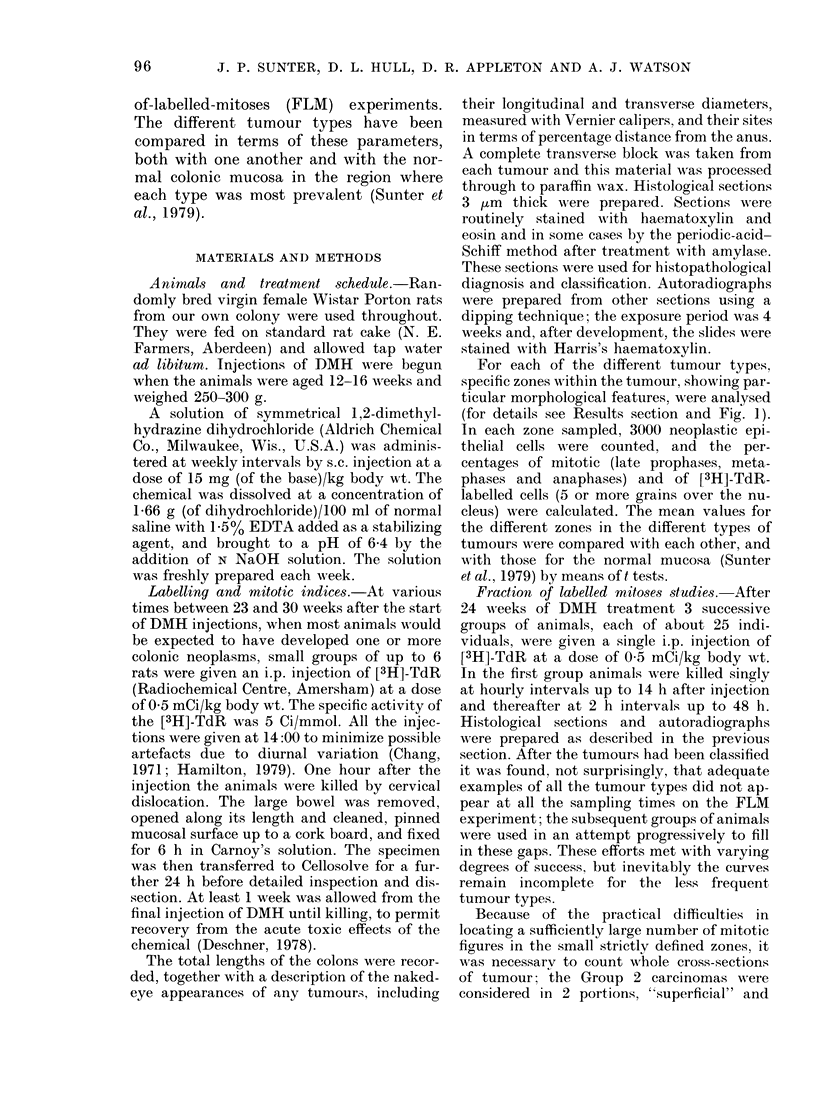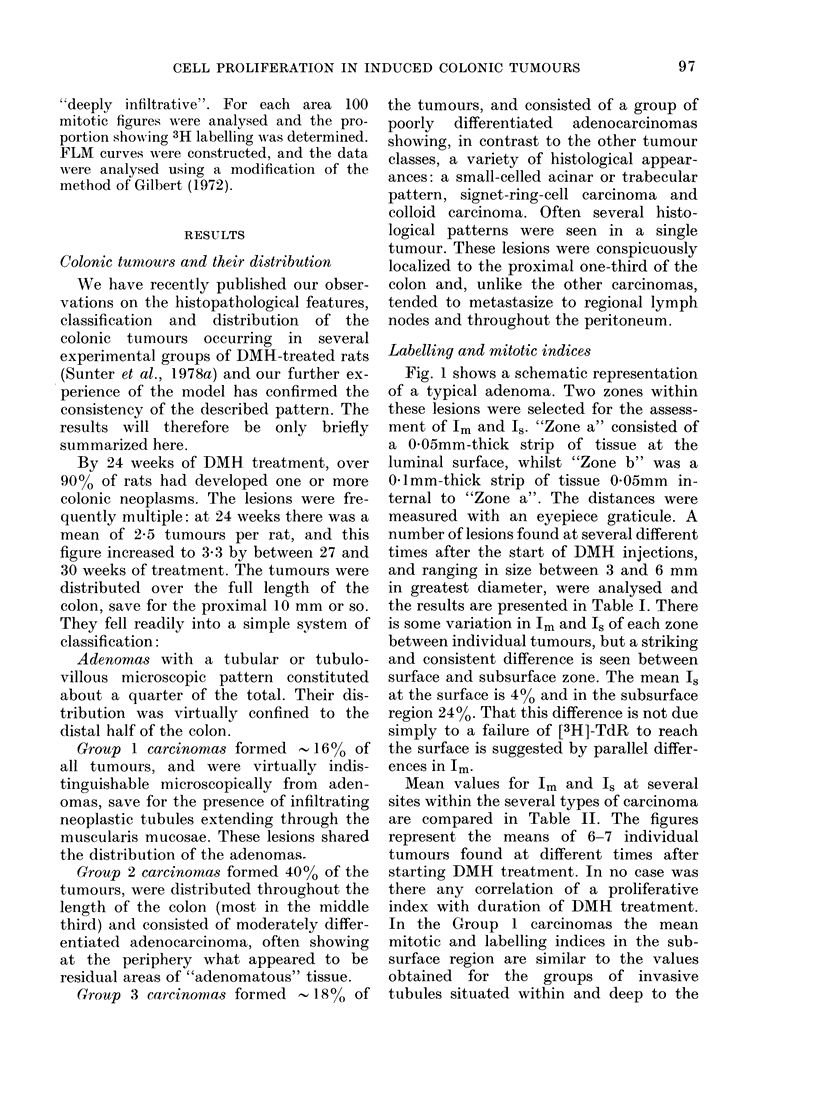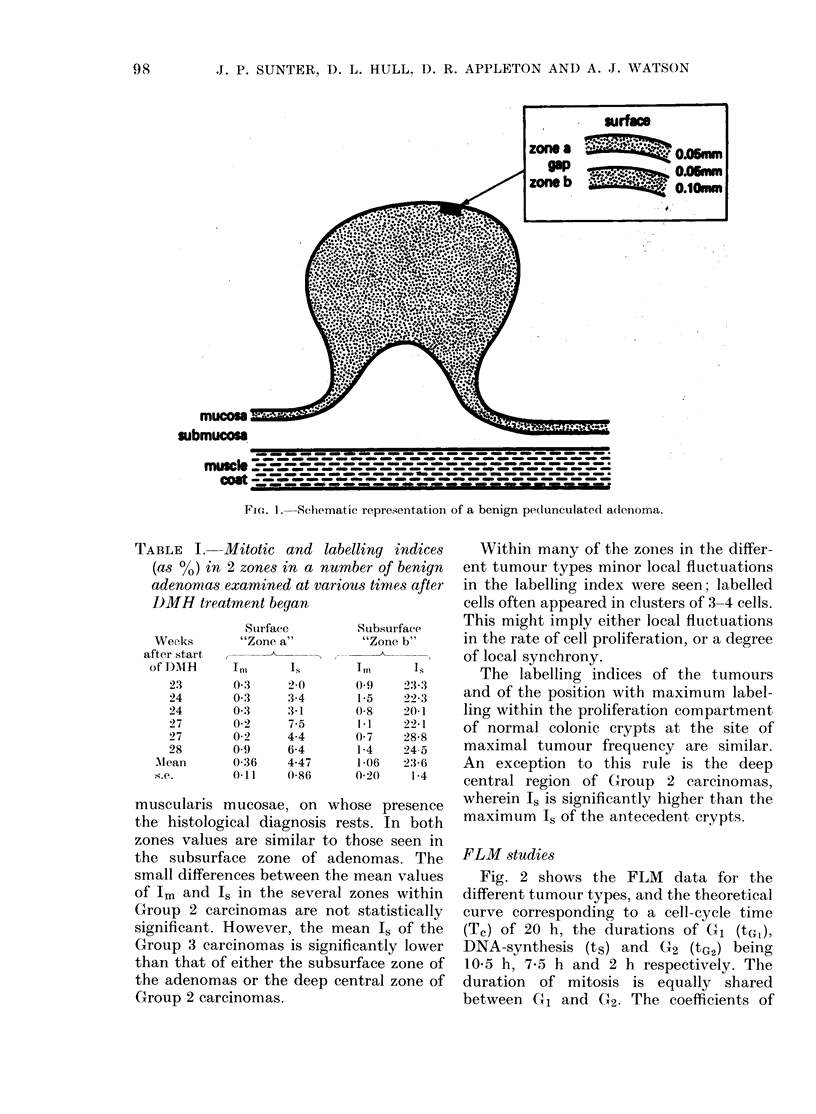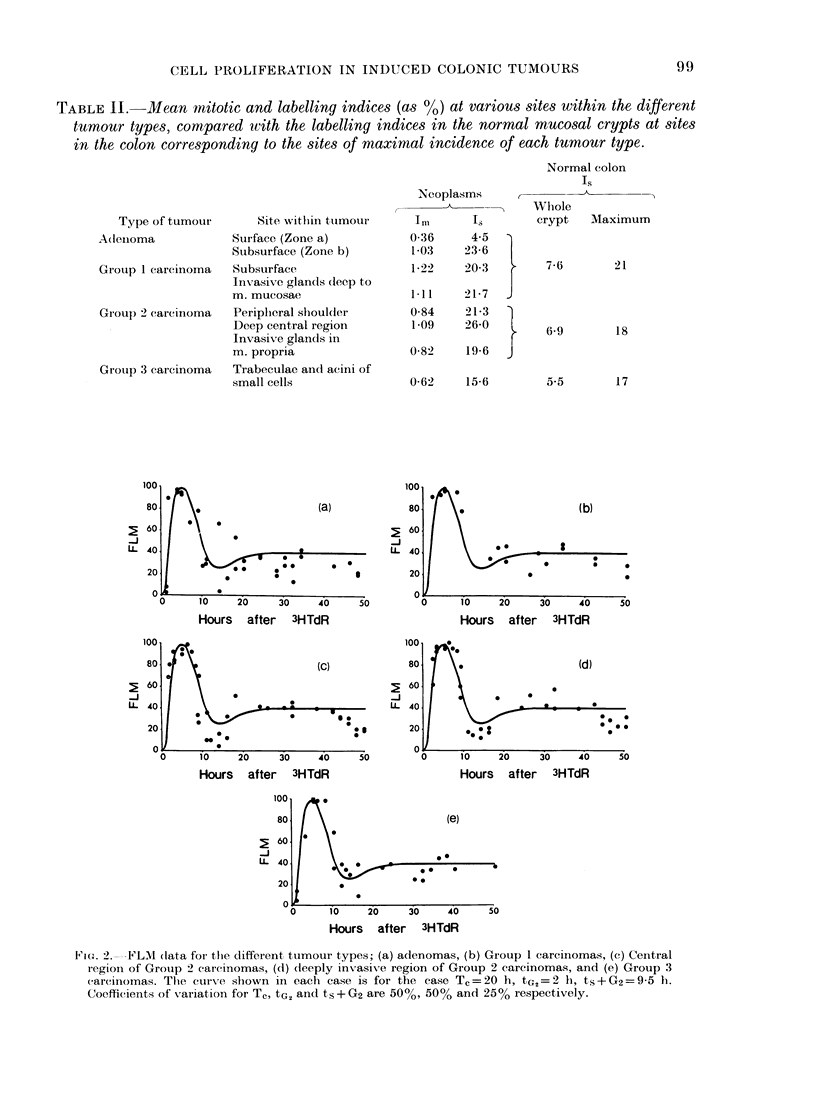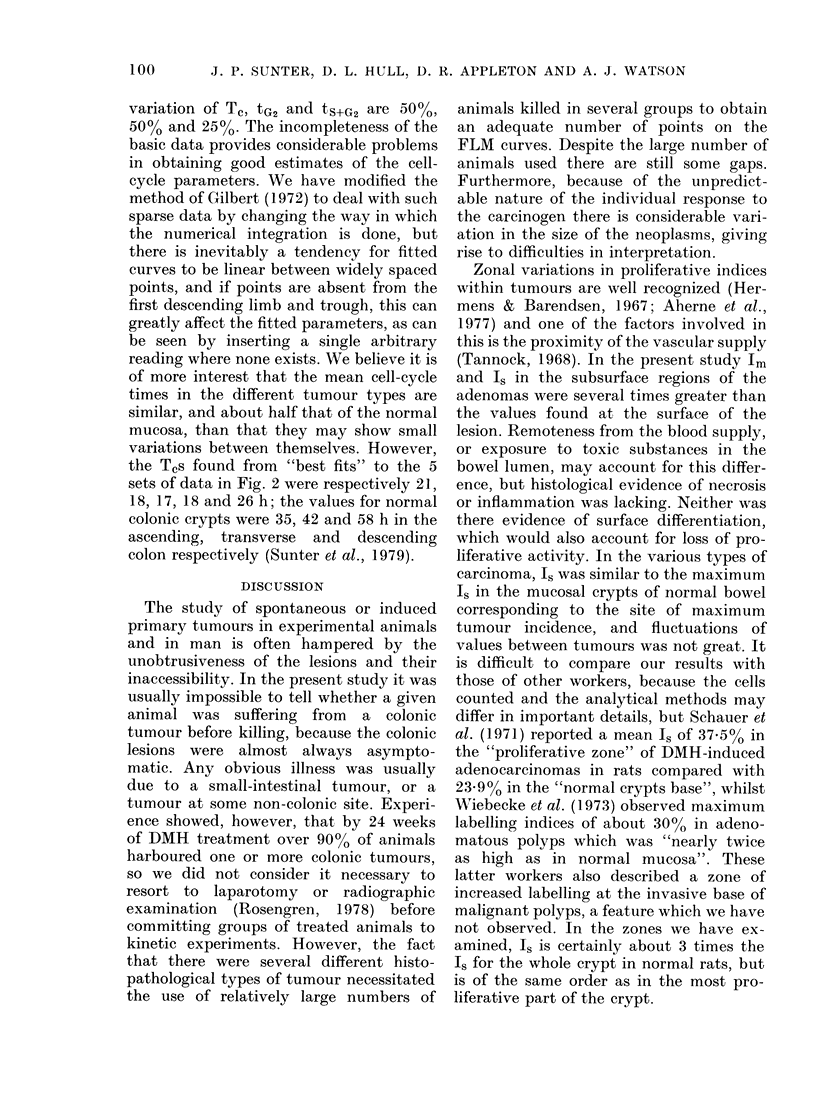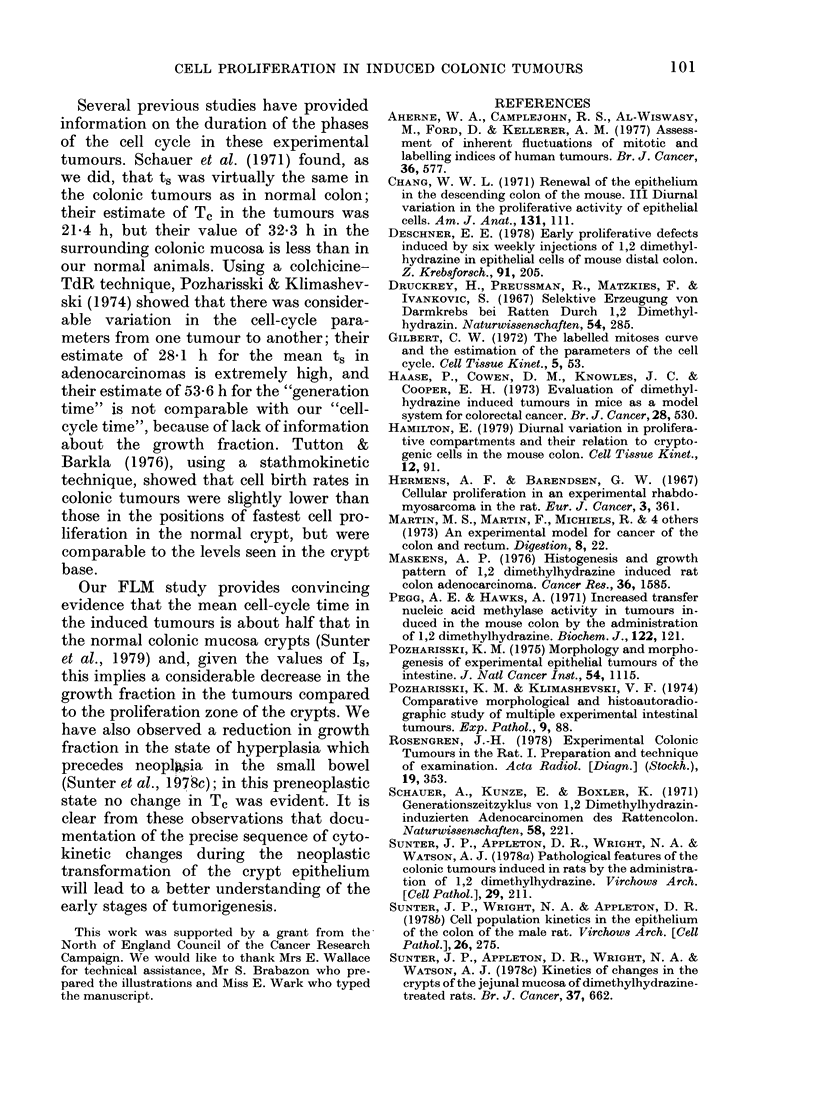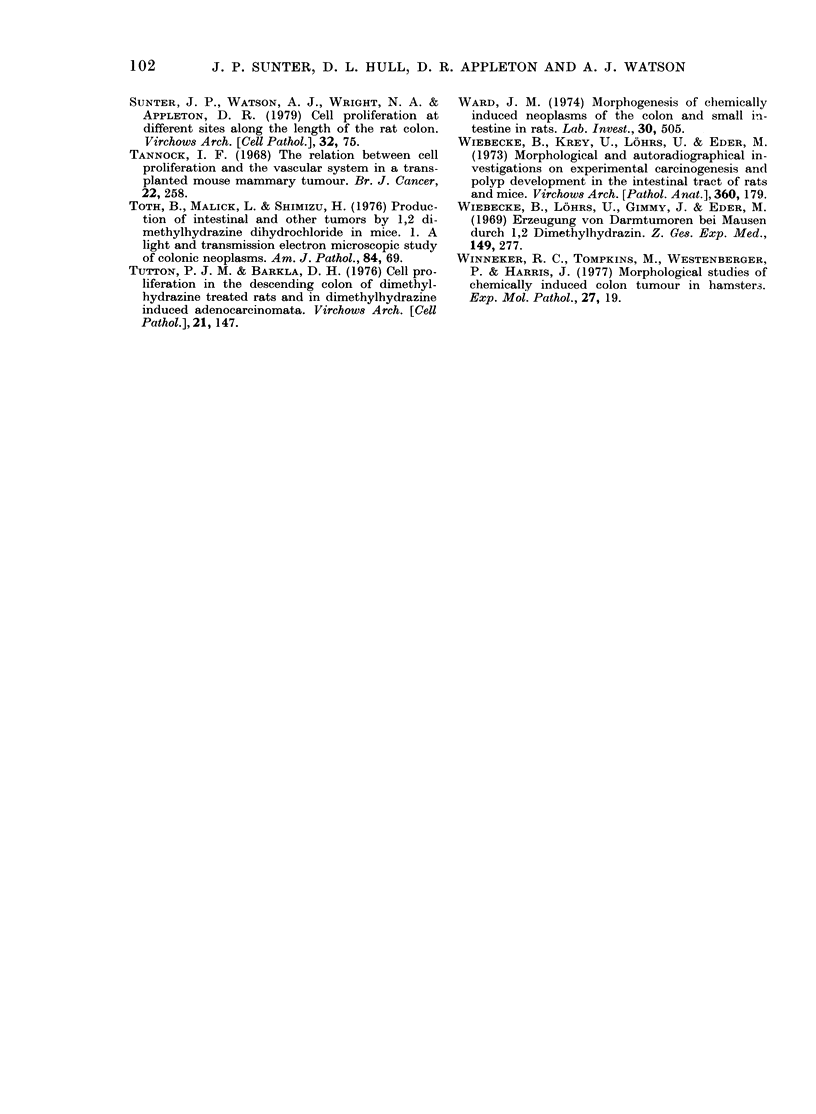# Cell proliferation of colonic neoplasms in dimethylhydrazine-treated rats.

**DOI:** 10.1038/bjc.1980.207

**Published:** 1980-07

**Authors:** J. P. Sunter, D. L. Hull, D. R. Appleton, A. J. Watson

## Abstract

We have measured mitotic indices and 3H-thymidine-labelling indices for the colonic epithelial tumours induced in rats by the administration of dimethyl-hydrazine (DMH). The fraction-of-labelled-mitoses (FLM) technique has been used to estimate the duration of the cell-cycle phases. In general, mitotic and labelling indices in the tumours are similar to those in the proliferation zone of the normal crypt epithelium; lesions considered to be least well differentiated on histological grounds appear to have the lowest mean labelling index. Benign tumours and the different types of malignant tumours have mean cell-cycle times about half those of the normal mucosa.


*Br. J. Cancer* (1980) **42**, 95

### CELL PROLIFERATION OF COLONIC NEOPLASMS IN DIMETHYLHYDRAZINE-TREATED RATS

J. P. SUNTER*, D. L. HULL†, D. R. APPLETON† AND A. J. WATSON*

*From the Departments of *Pathology and †Medical Statistics, University of Newcastle upon Tyne*

Received 8 February 1980 Accepted 20 March 1980

**Summary.—We have measured mitotic indices and $^3$H-thymidine-labelling indices for the colonic epithelial tumours induced in rats by the administration of dimethylhydrazine (DMH). The fraction-of-labelled-mitoses (FLM) technique has been used to estimate the duration of the cell-cycle phases.**

**In general, mitotic and labelling indices in the tumours are similar to those in the proliferation zone of the normal crypt epithelium; lesions considered to be least well differentiated on histological grounds appear to have the lowest mean labelling index. Benign tumours and the different types of malignant tumours have mean cell-cycle times about half those of the normal mucosa.**

THE PARENTERAL ADMINISTRATION of symmetrical 1,2-dimethylhydrazine (DMH) to several species of rodents causes the development of a number of different types of neoplasm. Tumours of the intestinal tract are particularly frequent, and this striking organotropism, first commented upon by Druckrey *et al.* (1967) in their studies on rats, has been amply confirmed by many other workers, both in rats (Wiebecke *et al.*, 1973; Martin *et al.*, 1973; Ward, 1974; Pozharisski, 1975) in mice (Wiebecke *et al.*, 1969; Pegg & Hawks, 1971; Haase *et al.*, 1973; Toth *et al.*, 1976) and in hamsters (Winneker *et al.*, 1977). The induced intestinal tumours are almost always of epithelial origin. In rats, tumours occur both in the small intestine and in the colon, whereas in mice the colonic neoplasms predominate overwhelmingly. It is as a highly selective colonic carcinogen that DMH has found most of its experimental applications.

Several reports have described in some detail the range of histopathological types of colonic tumours arising in DMH-treated rats (Wiebecke *et al.*, 1973; Ward, 1974; Pozharisski, 1975; Maskens, 1976). We have recently described a simple morphological classification of these different tumour types (Sunter *et al.*, 1978*a*) which are strikingly similar to the colonic neoplasms in humans. In the rat model, however, purely villous adenomas are never seen, and carcinomatous neoplasms outnumber adenomatous neoplasms by about 3 to 1. An interesting finding is that each tumour type has a characteristic distribution along the length of the rat colon, with the least-differentiated carcinomas concentrated proximally, whilst adenomas occur mainly in the distal half. To a certain extent these differences in distribution mirror the variation in morphology and kinetic organization of the rat colonic mucosa from one site to another (Sunter *et al.*, 1978*b*).

In the present study we have examined cell proliferation in the various types of colonic tumour arising in DMH-treated rats. Techniques used have included calculation of the mitotic index ($I_m$) and the $^3$H-thymidine ([$^3$H]-TdR)-labelling index ($I_s$), together with estimation of the major cell-cycle parameters from fraction-

Address for correspondence: Dr J. P. Sunter, Department of Pathology, Royal Victoria Infirmary, Queen Victoria Road, Newcastle upon Tyne NE1 4LP.

of-labelled-mitoses (FLM) experiments. The different tumour types have been compared in terms of these parameters, both with one another and with the normal colonic mucosa in the region where each type was most prevalent (Sunter *et al.*, 1979).

### MATERIALS AND METHODS

*Animals and treatment schedule.*—Randomly bred virgin female Wistar Porton rats from our own colony were used throughout. They were fed on standard rat cake (N. E. Farmers, Aberdeen) and allowed tap water *ad libitum.* Injections of DMH were begun when the animals were aged 12–16 weeks and weighed 250–300 g.

A solution of symmetrical 1,2-dimethylhydrazine dihydrochloride (Aldrich Chemical Co., Milwaukee, Wis., U.S.A.) was administered at weekly intervals by s.c. injection at a dose of 15 mg (of the base)/kg body wt. The chemical was dissolved at a concentration of 1·66 g (of dihydrochloride)/100 ml of normal saline with 1·5% EDTA added as a stabilizing agent, and brought to a pH of 6·4 by the addition of N NaOH solution. The solution was freshly prepared each week.

*Labelling and mitotic indices.*—At various times between 23 and 30 weeks after the start of DMH injections, when most animals would be expected to have developed one or more colonic neoplasms, small groups of up to 6 rats were given an i.p. injection of [$^3$H]-TdR (Radiochemical Centre, Amersham) at a dose of 0·5 mCi/kg body wt. The specific activity of the [$^3$H]-TdR was 5 Ci/mmol. All the injections were given at 14:00 to minimize possible artefacts due to diurnal variation (Chang, 1971; Hamilton, 1979). One hour after the injection the animals were killed by cervical dislocation. The large bowel was removed, opened along its length and cleaned, pinned mucosal surface up to a cork board, and fixed for 6 h in Carnoy's solution. The specimen was then transferred to Cellosolve for a further 24 h before detailed inspection and dissection. At least 1 week was allowed from the final injection of DMH until killing, to permit recovery from the acute toxic effects of the chemical (Deschner, 1978).

The total lengths of the colons were recorded, together with a description of the naked-eye appearances of any tumours, including their longitudinal and transverse diameters, measured with Vernier calipers, and their sites in terms of percentage distance from the anus. A complete transverse block was taken from each tumour and this material was processed through to paraffin wax. Histological sections 3 $\mu$m thick were prepared. Sections were routinely stained with haematoxylin and eosin and in some cases by the periodic-acid–Schiff method after treatment with amylase. These sections were used for histopathological diagnosis and classification. Autoradiographs were prepared from other sections using a dipping technique; the exposure period was 4 weeks and, after development, the slides were stained with Harris's haematoxylin.

For each of the different tumour types, specific zones within the tumour, showing particular morphological features, were analysed (for details see Results section and Fig. 1). In each zone sampled, 3000 neoplastic epithelial cells were counted, and the percentages of mitotic (late prophases, metaphases and anaphases) and of [$^3$H]-TdR-labelled cells (5 or more grains over the nucleus) were calculated. The mean values for the different zones in the different types of tumours were compared with each other, and with those for the normal mucosa (Sunter *et al.*, 1979) by means of *t* tests.

*Fraction of labelled mitoses studies.*—After 24 weeks of DMH treatment 3 successive groups of animals, each of about 25 individuals, were given a single i.p. injection of [$^3$H]-TdR at a dose of 0·5 mCi/kg body wt. In the first group animals were killed singly at hourly intervals up to 14 h after injection and thereafter at 2 h intervals up to 48 h. Histological sections and autoradiographs were prepared as described in the previous section. After the tumours had been classified it was found, not surprisingly, that adequate examples of all the tumour types did not appear at all the sampling times on the FLM experiment; the subsequent groups of animals were used in an attempt progressively to fill in these gaps. These efforts met with varying degrees of success, but inevitably the curves remain incomplete for the less frequent tumour types.

Because of the practical difficulties in locating a sufficiently large number of mitotic figures in the small strictly defined zones, it was necessary to count whole cross-sections of tumour; the Group 2 carcinomas were considered in 2 portions, "superficial" and

"deeply infiltrative". For each area 100 mitotic figures were analysed and the proportion showing $^3$H labelling was determined. FLM curves were constructed, and the data were analysed using a modification of the method of Gilbert (1972).

### RESULTS

### *Colonic tumours and their distribution*

We have recently published our observations on the histopathological features, classification and distribution of the colonic tumours occurring in several experimental groups of DMH-treated rats (Sunter *et al.*, 1978*a*) and our further experience of the model has confirmed the consistency of the described pattern. The results will therefore be only briefly summarized here.

By 24 weeks of DMH treatment, over 90% of rats had developed one or more colonic neoplasms. The lesions were frequently multiple: at 24 weeks there was a mean of 2·5 tumours per rat, and this figure increased to 3·3 by between 27 and 30 weeks of treatment. The tumours were distributed over the full length of the colon, save for the proximal 10 mm or so. They fell readily into a simple system of classification:

*Adenomas* with a tubular or tubulovillous microscopic pattern constituted about a quarter of the total. Their distribution was virtually confined to the distal half of the colon.

*Group* 1 *carcinomas* formed ~16% of all tumours, and were virtually indistinguishable microscopically from adenomas, save for the presence of infiltrating neoplastic tubules extending through the muscularis mucosae. These lesions shared the distribution of the adenomas.

*Group* 2 *carcinomas* formed 40% of the tumours, were distributed throughout the length of the colon (most in the middle third) and consisted of moderately differentiated adenocarcinoma, often showing at the periphery what appeared to be residual areas of "adenomatous" tissue.

*Group* 3 *carcinomas* formed ~18% of the tumours, and consisted of a group of poorly differentiated adenocarcinomas showing, in contrast to the other tumour classes, a variety of histological appearances: a small-celled acinar or trabecular pattern, signet-ring-cell carcinoma and colloid carcinoma. Often several histological patterns were seen in a single tumour. These lesions were conspicuously localized to the proximal one-third of the colon and, unlike the other carcinomas, tended to metastasize to regional lymph nodes and throughout the peritoneum.

### *Labelling and mitotic indices*

Fig. 1 shows a schematic representation of a typical adenoma. Two zones within these lesions were selected for the assessment of $I_m$ and $I_s$. "Zone a" consisted of a 0·05mm-thick strip of tissue at the luminal surface, whilst "Zone b" was a 0·1mm-thick strip of tissue 0·05mm internal to "Zone a". The distances were measured with an eyepiece graticule. A number of lesions found at several different times after the start of DMH injections, and ranging in size between 3 and 6 mm in greatest diameter, were analysed and the results are presented in Table I. There is some variation in $I_m$ and $I_s$ of each zone between individual tumours, but a striking and consistent difference is seen between surface and subsurface zone. The mean $I_s$ at the surface is 4% and in the subsurface region 24%. That this difference is not due simply to a failure of [$^3$H]-TdR to reach the surface is suggested by parallel differences in $I_m$.

Mean values for $I_m$ and $I_s$ at several sites within the several types of carcinoma are compared in Table II. The figures represent the means of 6–7 individual tumours found at different times after starting DMH treatment. In no case was there any correlation of a proliferative index with duration of DMH treatment. In the Group 1 carcinomas the mean mitotic and labelling indices in the subsurface region are similar to the values obtained for the groups of invasive tubules situated within and deep to the

FIG. 1.—Schematic representation of a benign pedunculated adenoma.

TABLE I.—*Mitotic and labelling indices (as %) in 2 zones in a number of benign adenomas examined at various times after DMH treatment began*

| Weeks after start of DMH | Surface "Zone a" $I_m$ | $I_s$ | Subsurface "Zone b" $I_m$ | $I_s$ |
|---|---|---|---|---|
| 23 | 0·3 | 2·0 | 0·9 | 23·3 |
| 24 | 0·3 | 3·4 | 1·5 | 22·3 |
| 24 | 0·3 | 3·1 | 0·8 | 20·1 |
| 27 | 0·2 | 7·5 | 1·1 | 22·1 |
| 27 | 0·2 | 4·4 | 0·7 | 28·8 |
| 28 | 0·9 | 6·4 | 1·4 | 24·5 |
| Mean | 0·36 | 4·47 | 1·06 | 23·6 |
| s.e. | 0·11 | 0·86 | 0·20 | 1·4 |

muscularis mucosae, on whose presence the histological diagnosis rests. In both zones values are similar to those seen in the subsurface zone of adenomas. The small differences between the mean values of $I_m$ and $I_s$ in the several zones within Group 2 carcinomas are not statistically significant. However, the mean $I_s$ of the Group 3 carcinomas is significantly lower than that of either the subsurface zone of the adenomas or the deep central zone of Group 2 carcinomas.

Within many of the zones in the different tumour types minor local fluctuations in the labelling index were seen; labelled cells often appeared in clusters of 3–4 cells. This might imply either local fluctuations in the rate of cell proliferation, or a degree of local synchrony.

The labelling indices of the tumours and of the position with maximum labelling within the proliferation compartment of normal colonic crypts at the site of maximal tumour frequency are similar. An exception to this rule is the deep central region of Group 2 carcinomas, wherein $I_s$ is significantly higher than the maximum $I_s$ of the antecedent crypts.

### *FLM studies*

Fig. 2 shows the FLM data for the different tumour types, and the theoretical curve corresponding to a cell-cycle time ($T_c$) of 20 h, the durations of $G_1$ ($t_{G_1}$), DNA-synthesis ($t_S$) and $G_2$ ($t_{G_2}$) being 10·5 h, 7·5 h and 2 h respectively. The duration of mitosis is equally shared between $G_1$ and $G_2$. The coefficients of

TABLE II.—*Mean mitotic and labelling indices (as %) at various sites within the different tumour types, compared with the labelling indices in the normal mucosal crypts at sites in the colon corresponding to the sites of maximal incidence of each tumour type.*

| | | Neoplasms | | Normal colon $I_s$ | |
|---|---|---|---|---|---|
| Type of tumour | Site within tumour | $I_m$ | $I_s$ | Whole crypt | Maximum |
| Adenoma | Surface (Zone a) | 0·36 | 4·5 | | |
| | Subsurface (Zone b) | 1·03 | 23·6 | | |
| Group 1 carcinoma | Subsurface | 1·22 | 20·3 | 7·6 | 21 |
| | Invasive glands deep to m. mucosae | 1·11 | 21·7 | | |
| Group 2 carcinoma | Peripheral shoulder | 0·84 | 21·3 | | |
| | Deep central region | 1·09 | 26·0 | 6·9 | 18 |
| | Invasive glands in m. propria | 0·82 | 19·6 | | |
| Group 3 carcinoma | Trabeculae and acini of small cells | 0·62 | 15·6 | 5·5 | 17 |

FIG. 2.—FLM data for the different tumour types; (a) adenomas, (b) Group 1 carcinomas, (c) Central region of Group 2 carcinomas, (d) deeply invasive region of Group 2 carcinomas, and (e) Group 3 carcinomas. The curve shown in each case is for the case $T_c = 20$ h, $t_{G_2} = 2$ h, $t_S + G_2 = 9·5$ h. Coefficients of variation for $T_c$, $t_{G_2}$ and $t_S + G_2$ are 50%, 50% and 25% respectively.

variation of $T_c$, $t_{G_2}$ and $t_{S+G_2}$ are 50%, 50% and 25%. The incompleteness of the basic data provides considerable problems in obtaining good estimates of the cell-cycle parameters. We have modified the method of Gilbert (1972) to deal with such sparse data by changing the way in which the numerical integration is done, but there is inevitably a tendency for fitted curves to be linear between widely spaced points, and if points are absent from the first descending limb and trough, this can greatly affect the fitted parameters, as can be seen by inserting a single arbitrary reading where none exists. We believe it is of more interest that the mean cell-cycle times in the different tumour types are similar, and about half that of the normal mucosa, than that they may show small variations between themselves. However, the $T_c$s found from "best fits" to the 5 sets of data in Fig. 2 were respectively 21, 18, 17, 18 and 26 h; the values for normal colonic crypts were 35, 42 and 58 h in the ascending, transverse and descending colon respectively (Sunter *et al.*, 1979).

### DISCUSSION

The study of spontaneous or induced primary tumours in experimental animals and in man is often hampered by the unobtrusiveness of the lesions and their inaccessibility. In the present study it was usually impossible to tell whether a given animal was suffering from a colonic tumour before killing, because the colonic lesions were almost always asymptomatic. Any obvious illness was usually due to a small-intestinal tumour, or a tumour at some non-colonic site. Experience showed, however, that by 24 weeks of DMH treatment over 90% of animals harboured one or more colonic tumours, so we did not consider it necessary to resort to laparotomy or radiographic examination (Rosengren, 1978) before committing groups of treated animals to kinetic experiments. However, the fact that there were several different histopathological types of tumour necessitated the use of relatively large numbers of animals killed in several groups to obtain an adequate number of points on the FLM curves. Despite the large number of animals used there are still some gaps. Furthermore, because of the unpredictable nature of the individual response to the carcinogen there is considerable variation in the size of the neoplasms, giving rise to difficulties in interpretation.

Zonal variations in proliferative indices within tumours are well recognized (Hermens & Barendsen, 1967; Aherne *et al.*, 1977) and one of the factors involved in this is the proximity of the vascular supply (Tannock, 1968). In the present study $I_m$ and $I_s$ in the subsurface regions of the adenomas were several times greater than the values found at the surface of the lesion. Remoteness from the blood supply, or exposure to toxic substances in the bowel lumen, may account for this difference, but histological evidence of necrosis or inflammation was lacking. Neither was there evidence of surface differentiation, which would also account for loss of proliferative activity. In the various types of carcinoma, $I_s$ was similar to the maximum $I_s$ in the mucosal crypts of normal bowel corresponding to the site of maximum tumour incidence, and fluctuations of values between tumours was not great. It is difficult to compare our results with those of other workers, because the cells counted and the analytical methods may differ in important details, but Schauer *et al.* (1971) reported a mean $I_s$ of 37·5% in the "proliferative zone" of DMH-induced adenocarcinomas in rats compared with 23·9% in the "normal crypts base", whilst Wiebecke *et al.* (1973) observed maximum labelling indices of about 30% in adenomatous polyps which was "nearly twice as high as in normal mucosa". These latter workers also described a zone of increased labelling at the invasive base of malignant polyps, a feature which we have not observed. In the zones we have examined, $I_s$ is certainly about 3 times the $I_s$ for the whole crypt in normal rats, but is of the same order as in the most proliferative part of the crypt.

Several previous studies have provided information on the duration of the phases of the cell cycle in these experimental tumours. Schauer *et al.* (1971) found, as we did, that $t_S$ was virtually the same in the colonic tumours as in normal colon; their estimate of $T_C$ in the tumours was 21·4 h, but their value of 32·3 h in the surrounding colonic mucosa is less than in our normal animals. Using a colchicine–TdR technique, Pozharisski & Klimashevski (1974) showed that there was considerable variation in the cell-cycle parameters from one tumour to another; their estimate of 28·1 h for the mean $t_S$ in adenocarcinomas is extremely high, and their estimate of 53·6 h for the "generation time" is not comparable with our "cell-cycle time", because of lack of information about the growth fraction. Tutton & Barkla (1976), using a stathmokinetic technique, showed that cell birth rates in colonic tumours were slightly lower than those in the positions of fastest cell proliferation in the normal crypt, but were comparable to the levels seen in the crypt base.

Our FLM study provides convincing evidence that the mean cell-cycle time in the induced tumours is about half that in the normal colonic mucosa crypts (Sunter *et al.*, 1979) and, given the values of $I_S$, this implies a considerable decrease in the growth fraction in the tumours compared to the proliferation zone of the crypts. We have also observed a reduction in growth fraction in the state of hyperplasia which precedes neoplasia in the small bowel (Sunter *et al.*, 1978*c*); in this preneoplastic state no change in $T_C$ was evident. It is clear from these observations that documentation of the precise sequence of cytokinetic changes during the neoplastic transformation of the crypt epithelium will lead to a better understanding of the early stages of tumorigenesis.

This work was supported by a grant from the North of England Council of the Cancer Research Campaign. We would like to thank Mrs E. Wallace for technical assistance, Mr S. Brabazon who prepared the illustrations and Miss E. Wark who typed the manuscript.

### REFERENCES

AHERNE, W. A., CAMPLEJOHN, R. S., AL-WISWASY, M., FORD, D. & KELLERER, A. M. (1977) Assessment of inherent fluctuations of mitotic and labelling indices of human tumours. *Br. J. Cancer*, **36**, 577.

CHANG, W. W. L. (1971) Renewal of the epithelium in the descending colon of the mouse. III Diurnal variation in the proliferative activity of epithelial cells. *Am. J. Anat.*, **131**, 111.

DESCHNER, E. E. (1978) Early proliferative defects induced by six weekly injections of 1,2 dimethylhydrazine in epithelial cells of mouse distal colon. *Z. Krebsforsch.*, **91**, 205.

DRUCKREY, H., PREUSSMAN, R., MATZKIES, F. & IVANKOVIC, S. (1967) Selektive Erzeugung von Darmkrebs bei Ratten Durch 1,2 Dimethylhydrazin. *Naturwissenschaften*, **54**, 285.

GILBERT, C. W. (1972) The labelled mitoses curve and the estimation of the parameters of the cell cycle. *Cell Tissue Kinet.*, **5**, 53.

HAASE, P., COWEN, D. M., KNOWLES, J. C. & COOPER, E. H. (1973) Evaluation of dimethylhydrazine induced tumours in mice as a model system for colorectal cancer. *Br. J. Cancer*, **28**, 530.

HAMILTON, E. (1979) Diurnal variation in proliferative compartments and their relation to cryptogenic cells in the mouse colon. *Cell Tissue Kinet.*, **12**, 91.

HERMENS, A. F. & BARENDSEN, G. W. (1967) Cellular proliferation in an experimental rhabdomyosarcoma in the rat. *Eur. J. Cancer*, **3**, 361.

MARTIN, M. S., MARTIN, F., MICHIELS, R. & 4 others (1973) An experimental model for cancer of the colon and rectum. *Digestion*, **8**, 22.

MASKENS, A. P. (1976) Histogenesis and growth pattern of 1,2 dimethylhydrazine induced rat colon adenocarcinoma. *Cancer Res.*, **36**, 1585.

PEGG, A. E. & HAWKS, A. (1971) Increased transfer nucleic acid methylase activity in tumours induced in the mouse colon by the administration of 1,2 dimethylhydrazine. *Biochem. J.*, **122**, 121.

POZHARISSKI, K. M. (1975) Morphology and morphogenesis of experimental epithelial tumours of the intestine. *J. Natl Cancer Inst.*, **54**, 1115.

POZHARISSKI, K. M. & KLIMASHEVSKI, V. F. (1974) Comparative morphological and histoautoradiographic study of multiple experimental intestinal tumours. *Exp. Pathol.*, **9**, 88.

ROSENGREN, J.-H. (1978) Experimental Colonic Tumours in the Rat. I. Preparation and technique of examination. *Acta Radiol.* [*Diagn.*] (*Stockh.*), **19**, 353.

SCHAUER, A., KUNZE, E. & BOXLER, K. (1971) Generationszeitzyklus von 1,2 Dimethylhydrazin-induzierten Adenocarcinomen des Rattencolon. *Naturwissenschaften*, **58**, 221.

SUNTER, J. P., APPLETON, D. R., WRIGHT, N. A. & WATSON, A. J. (1978*a*) Pathological features of the colonic tumours induced in rats by the administration of 1,2 dimethylhydrazine. *Virchows Arch.* [*Cell Pathol.*], **29**, 211.

SUNTER, J. P., WRIGHT, N. A. & APPLETON, D. R. (1978*b*) Cell population kinetics in the epithelium of the colon of the male rat. *Virchows Arch.* [*Cell Pathol.*], **26**, 275.

SUNTER, J. P., APPLETON, D. R., WRIGHT, N. A. & WATSON, A. J. (1978*c*) Kinetics of changes in the crypts of the jejunal mucosa of dimethylhydrazine-treated rats. *Br. J. Cancer*, **37**, 662.

SUNTER, J. P., WATSON, A. J., WRIGHT, N. A. & APPLETON, D. R. (1979) Cell proliferation at different sites along the length of the rat colon. *Virchows Arch.* [*Cell Pathol.*], **32**, 75.

TANNOCK, I. F. (1968) The relation between cell proliferation and the vascular system in a transplanted mouse mammary tumour. *Br. J. Cancer*, **22**, 258.

TOTH, B., MALICK, L. & SHIMIZU, H. (1976) Production of intestinal and other tumors by 1,2 dimethylhydrazine dihydrochloride in mice. 1. A light and transmission electron microscopic study of colonic neoplasms. *Am. J. Pathol.*, **84**, 69.

TUTTON, P. J. M. & BARKLA, D. H. (1976) Cell proliferation in the descending colon of dimethylhydrazine treated rats and in dimethylhydrazine induced adenocarcinomata. *Virchows Arch.* [*Cell Pathol.*], **21**, 147.

WARD, J. M. (1974) Morphogenesis of chemically induced neoplasms of the colon and small intestine in rats. *Lab. Invest.*, **30**, 505.

WIEBECKE, B., KREY, U., LÖHRS, U. & EDER, M. (1973) Morphological and autoradiographical investigations on experimental carcinogenesis and polyp development in the intestinal tract of rats and mice. *Virchows Arch.* [*Pathol. Anat.*], **360**, 179.

WIEBECKE, B., LÖHRS, U., GIMMY, J. & EDER, M. (1969) Erzeugung von Darmtumoren bei Mausen durch 1,2 Dimethylhydrazin. *Z. Ges. Exp. Med.*, **149**, 277.

WINNEKER, R. C., TOMPKINS, M., WESTENBERGER, P. & HARRIS, J. (1977) Morphological studies of chemically induced colon tumour in hamsters. *Exp. Mol. Pathol.*, **27**, 19.